# Draft Genome Sequence of *Mycobacterium abscessus* Bamboo

**DOI:** 10.1128/genomeA.00388-17

**Published:** 2017-05-18

**Authors:** Michelle Yee, David Klinzing, Jun-Rong Wei, Martin Gengenbacher, Eric J. Rubin, Thomas Dick

**Affiliations:** a Department of Microbiology and Immunology, Yong Loo Lin School of Medicine, National University of Singapore, Singapore, Republic of Singapore; b AITbiotech Singapore, Singapore, Republic of Singapore; c Department of Immunology and Infectious Diseases, Harvard T. H. Chan School of Public Health, Boston, Massachusetts, USA; d Department of Microbiology and Immunobiology, Harvard Medical School, Boston, Massachusetts, USA; e Public Health Research Institute, New Jersey Medical School, Rutgers, the State University of New Jersey, Newark, New Jersey, USA

## Abstract

*Mycobacterium abscessus*, an intrinsically multidrug-resistant pathogen, causes chronic incurable lung disease. New drugs for this emerging pathogen represent an urgent unmet medical need. Here, we report a draft genome sequence of *M. abscessus* Bamboo, a clinical isolate used as a screening strain for drug discovery.

## GENOME ANNOUNCEMENT

*Mycobacterium abscessus*, a fast-growing nontuberculous mycobacterium (NTM), is an emerging pathogen causing chronic pulmonary infections worldwide, particularly in cystic fibrosis (CF) patients, resulting in increased inflammatory lung damage, worse clinical outcome, and increased mortality (1–3). The intrinsic multidrug-resistant nature of *M. abscessus* and the lack of an established standard drug regimen significantly complicate chemotherapy (1, 4). To further exacerbate the situation, recent whole-genome analyses suggested the transmission of *M. abscessus* infection between CF patients (5, 6). With this pathogen rising to the level of a global threat, there is an urgent call to deploy funds for *M. abscessus* drug discovery enabling the development of new effective drugs (7). Here, we report the draft genome sequence of *M. abscessus* Bamboo, a clinical isolate used as a screening strain in our ongoing drug discovery initiative focusing on NTM (8, 9).

*M. abscessus* Bamboo was isolated from a sputum sample from a patient with amyotrophic lateral sclerosis and bronchiectasis and was provided by Wei-Chang Huang, Taichung Veterans General Hospital, Taichung, Taiwan. Based on the 16S rRNA as well as the *erm*(41) and *rpoB* gene sequences (10, 11), *M. abscessus* Bamboo was classified as a member of *M. abscessus* subsp. *abscessus*. When referenced to the *M. abscessus* type strain (ATCC 19977), it has 100% identity in 16S rRNA and 99% similarity in both the *erm*(41) and *rpoB* genes. With a wild-type 23S rRNA and T28C polymorphism in *erm*(41), it is phenotypically susceptible to clarithromycin (MIC_90_, 0.4 μM) and shows no inducible clarithromycin resistance (D. B. Aziz, J. L. Low, M.-L. Wu, M. Gengenbacher, J. W. P. Teo, V. Dartois, and T. Dick, unpublished data) (12).

*M. abscessus* Bamboo was cultured in Middlebrook 7H9 liquid broth at 37°C with agitation, pelleted, and heat-inactivated at 95°C. Genomic DNA was extracted by standard methods using phenol-chloroform. The DNA library preparation was performed using Covaris shearing and the Illumina TruSeq Nano DNA library preparation kit. Samples were sequenced using the Illumina MiSeq platform (AITbiotech, Singapore). Two sequencing runs of 2 × 300-bp read lengths were carried out, and reads from both runs were combined for analysis. A total of 5,580,026 paired reads were generated and quality checked using FastQC. Reads were quality trimmed using fqtrim (http://ccb.jhu.edu/software/fqtrim/) with a window size of 7, minimum average Q score of 28, and minimum posttrim length of 35. Following trimming, 5,277,626 paired-end reads were utilized for *de novo* assembly using SPAdes (version 3.6.2) (13), with k-mer sizes of 33, 55, 77, 99, and 127. A total of 43 contigs larger than 500 bp in length were generated, with the largest contig containing 659,675 bp, and an overall *N*_50_ of 308,472 bp. These contigs were annotated using the Rapid Annotations using Subsystems Technology (RAST) server (version 2) (14), with *M. abscessus* ATCC 19977^T^ (accession no. NC_010397.1) as a reference. This assembled draft genome is 5,118,280 bp long with 63.9% G+C content, predicted to contain 5,039 coding sequences and 49 RNAs (46 tRNA and 3 rRNA).

### Accession number(s).

This whole-genome shotgun project has been deposited at DDBJ/ENA/GenBank under the accession no. MVDX00000000. The version described in this paper is version MVDX01000000.
